# Antagonistic coevolution between hosts and sexually transmitted infections

**DOI:** 10.1111/evo.13883

**Published:** 2019-12-17

**Authors:** Ben Ashby

**Affiliations:** ^1^ Department of Mathematical Sciences University of Bath Bath BA2 7AY United Kingdom

**Keywords:** Fluctuating selection, host‐parasite, mate choice, parasite‐mediated sexual selection, STD, virulence

## Abstract

Sexually transmitted infections (STIs) are predicted to play an important role in the evolution of host mating strategies, and vice versa, yet our understanding of host‐STI coevolution is limited. Previous theoretical work has shown mate choice can evolve to prevent runaway STI virulence evolution in chronic, sterilizing infections. Here, I generalize this theory to examine how a broader range of life‐history traits influence coevolution; specifically, how host preferences for healthy mates and STI virulence coevolve when infections are acute and can cause mortality or sterility, and hosts do not form long‐term sexual partnerships. I show that mate choice reduces both mortality and sterility virulence, with qualitatively different outcomes depending on the mode of virulence, costs associated with mate choice, recovery rates, and host lifespan. For example, fluctuating selection—a key finding in previous work—is most likely when hosts have moderate lifespans, STIs cause sterility and long infections, and costs of mate choice are low. The results reveal new insights into the coevolution of mate choice and STI virulence as different life‐history traits vary, providing increased support for parasite‐mediated sexual selection as a potential driver of host mate choice, and mate choice as a constraint on the evolution of virulence.

Parasite‐mediated sexual selection (PMSS) is predicted to lead to the evolution of reproductive strategies that limit the risk of infection from mating (Hamilton and Zuk 1982; Sheldon 1993; Loehle 1997). By avoiding mates with signs of disease, organisms should be able to increase their reproductive success, either because they might choose partners possessing genes that confer resistance to disease (the “good genes” hypothesis; Hamilton and Zuk 1982) or simply because they choose mates that are currently uninfected and hence are a low‐risk option (the “transmission avoidance hypothesis”; Loehle 1997). Both hypotheses have been the subject of intense empirical research with varying evidence in support of and against PMSS (Borgia 1986; Borgia and Collis 1989; Clayton 1990, 1991; Hamilton and Poulin 1997; Abbot and Dill 2001; Webberley et al. 2002; Balenger and Zuk 2014; Jones et al. 2015; Ashby et al. 2019). Empirical studies have explored both sexually and nonsexually transmitted infections, but although one may intuitively expect sexual transmission to be the main driver of PMSS, this is not necessarily the case, and in theory both sexual and nonsexual transmissions may contribute to PMSS. In some cases, females have been found to prefer uninfected males—for example, Clayton (1990) found that female Rock Doves (*Columba livia*) prefer males without lice (which can be transmitted by physical contact during mating or otherwise, or by a vector), suggesting support for PMSS—whereas in other cases females appear unable to distinguish between infected males—for instance, female milkweed leaf beetles (*Labidomera clivicollis*; Abbot and Dill 2001) and two‐spot ladybirds (*Adalia bipunctata*; Webberley et al. 2002) do not avoid males with sexually transmitted mites.

In parallel, there has been much theoretical interest in understanding the role of parasites, especially sexually transmitted infections (STIs), in the evolution of host mating strategies, and the role of host mating behavior in the evolution of STIs (Thrall et al. 1997, 2000; Knell 1999; Boots and Knell 2002; Kokko et al. 2002; Ashby and Gupta 2013; McLeod and Day 2014; Ashby and Boots 2015). STIs are of particular interest as they are inherently tightly linked to host reproduction, unlike non‐STIs, and are more likely to have negative effects on host fecundity (Lockhart et al. 1996). This body of theoretical work has generally predicted that STIs may indeed act as a strong force of selection on host mating strategies.

Although changes in host mating behavior arising from PMSS will in turn affect STI evolution, forming a coevolutionary feedback, almost all theoretical studies only consider one‐sided adaptation of either the host or the STI. To date, it appears that only two theoretical studies have considered host‐STI coevolution. First, Ashby and Boots (2015) showed that the evolution of mate choice can prevent runaway selection for sterility virulence in STIs, leading to either stable levels of choosiness and virulence or coevolutionary cycling in these traits. Second, Wardlaw and Agrawal (2019) showed how mortality virulence escalates sexual conflict, whereas sterility virulence de‐escalates sexual conflict, thus showing how the mode of virulence can qualitatively change host‐STI coevolution. Together, these results represent important first steps in understanding host and STI coevolution, but we have only begun to scratch the surface. For example, Ashby and Boots (2015) focused on chronic, fertility‐reducing STIs in serially monogamous hosts, which are reasonable assumptions for many host species and STIs: for example, approximately 90% of bird species are thought to be monogamous (Kleiman 1977), and STIs often cause chronic infections, are more likely to cause reductions in fecundity, and typically have less of an impact on mortality than non‐STIs (Lockhart et al. 1996; Knell and Webberley 2004). Although serial monogamy is a useful place to start, many species do not form exclusive monogamous partnerships and instead carry out extra‐pair copulations or form no lasting sexual partnerships at all (Kleiman 1977; Forstmeier et al. 2014). Because many species are not serially monogamous, it is of particular interest how mate choice coevolves with STIs in other mating systems. For instance, if hosts form ephemeral rather than long‐term sexual partnerships, how and when will mate choice evolve? Although the importance of any given ephemeral sexual partnership is lower than under serial monogamy, the accumulation of many sexual partners over the lifetime of the host may still select for mate choice. In addition, many STIs are known to increase mortality—for example, HIV and syphilis in humans, and dourine in equines (Gizaw et al. 2017)—and to cause acute rather than chronic infections (e.g., *Chlamydia trachomatis* can be cleared by a number of mammalian species; Miyairi et al. 2010). Broadening our understanding of host‐STI coevolution therefore requires the development of theory that captures alternative host mating systems and disease outcomes.

Here, I examine a simple model of host‐STI coevolution when sexual partnerships are short term (ephemeral) and disease causes variable mortality or sterility virulence, and for different recovery rates. Using evolutionary invasion analysis, I first show how mate choice leads to lower optimal levels of mortality and sterility virulence. I then show how and when mate choice is likely to evolve under different disease characteristics. Finally, I consider host‐STI coevolution, showing that coevolutionary cycling is typically more common under sterility virulence, whereas mortality virulence tends to lead to more stable outcomes. I also identify conditions when polymorphism in host mate choice can evolve through evolutionary branching, but this outcome only occurs under a narrow set of conditions. Finally, I examine how costs associated with mate choice, the rate of recovery from infection, and the lifespan of the host impact host‐STI coevolution. Combined with previous studies, these results show that PMSS can occur for a broad range of host and STI life‐history traits.

## Methods

I model the dynamics of an STI in a well‐mixed host population, which for simplicity I treat as a single hermaphroditic sex as there is assumed to be no sex‐specific variation in disease characteristics. The epidemiological and mating dynamics of a one‐host‐one‐STI system are described by

(1) $$\frac{\mathrm{dS}}{\mathrm{dt}}=\underset{\mathrm{births}}{\underset{︸}{b(f,g,v)}}-\underset{\mathrm{infection}}{\underset{︸}{\beta [\mathrm{SI}]}}-\underset{\text{natural}\;\text{mortality}}{\underset{︸}{\mathrm{dS}}}+\underset{\mathrm{recovery}}{\underset{︸}{\gamma I}},$$

(2) $$\frac{\mathrm{dI}}{\mathrm{dt}}=\underset{\mathrm{infection}}{\underset{︸}{\beta [\mathrm{SI}]}}-\underset{\mathrm{natural}\;\mathrm{mortality}}{\underset{︸}{\mathrm{dI}}}-\underset{\mathrm{mortality}\;\mathrm{virulence}}{\underset{︸}{\alpha I}}-\underset{\mathrm{recovery}}{\underset{︸}{\gamma I}}\;,$$

where *S* and *I* are the densities of susceptible and infected individuals, respectively; b(f,g,v) is the host birth rate, which depends on the fecundity of infected hosts relative to uninfected hosts, *f*, with 0≤f≤1, the strength of mate choice (i.e., how strongly individuals prefer uninfected mates), g≥0, and *v*, which is used to relate the mode of virulence to mate choice (defined below); *d* is the natural mortality rate; α is the disease‐associated mortality rate; β is the transmission probability per sexual contact; γ is the recovery rate; and [XY] is the mating rate between individuals in classes X∈{S,I} and Y∈{S,I} (see Table 1 for full list of parameters and variables). Hereafter, I assume that mortality virulence and sterility virulence may be functions of transmissibility (i.e., α=α(β), f=f(β)) as parasites may need to damage their hosts or use host resources to produce transmission stages, and the more transmission stages produced the greater the damage is likely to be to the host (see Alizon et al. 2009 and Acevedo et al. 2019 for discussions of the transmission‐virulence trade‐off hypothesis). For simplicity, I assume linear functions to control the relationships between transmission and virulence such that f(β)=1−ηβ for ηβ<1 and 0 otherwise for sterility virulence, and α(β)=κβ for mortality virulence, with η and κ parameters that define the strength of these relationships. Such functions correspond to a situation where the damage caused to the host is proportional to the number of transmission stages produced by the STI. I restrict my analysis to how one mode of virulence at most varies and affects mate choice by setting η=0 and/or κ=0 and by using v=v(β) to relate the current mode of virulence to mate choice, with v(β)=1−f(β) in the case of sterility virulence and v(β)=α(β)/κ in the case of mortality virulence.

**Table 1:** Description of parameters and variables. The variables for the polymorphic model (equations 12–13) are identical except for the addition of subscripts

Parameter/variable	Description
b(f(β),g,v(β))	Host birth rate
***d***	Natural mortality rate
***f***(***β***)	Fecundity of infected hosts relative to uninfected hosts, which may be a function of transmission
***g***	Strength of mate choice
***h***	Strength of density‐dependent competition
***m***_***S***_(***g***)	Probability of accepting an uninfected mate
***m***_***I***_(***g***, ***v***(***β***))	Probability of accepting an infected mate
***p***	Baseline per‐capita mating rate
***r***	Maximum reproduction rate per mating
***v***(***β***)	The impact of STI virulence on mate choice
***R***_0_(***g***, ***β***)	Basic reproductive ratio
***S***, ***I***	Density of susceptible and infected hosts, respectively
***N***	Total density of hosts
[***XY***]	Sexual contact rate between hosts in the *X* and *Y* classes
[***XY***]_***b***_	Contribution to births from hosts in the *X* and *Y* classes in the polymorphic model
***S***_○_	Sum over all susceptible hosts in the polymorphic model
***I***_***i***○_	Sum over all parasite types in hosts of type *i* in the polymorphic model
***I***_○***j***_	Sum over all host types infected with parasites of type *j* in the polymorphic model
$I_{\circ \circ }$	Sum over all infected hosts in the polymorphic model
α(β)	Mortality virulence, which may be a function of transmission
***β***	Transmission probability per sexual contact
***γ***	Recovery rate
***ζ***	Strength of host costs
***η***	Strength of transmission‐sterility virulence relationship
***κ***	Strength of transmission‐mortality virulence relationship
***n***_***h***_, ***n***_***p***_	Number of host and parasite types, respectively, in the polymorphic model

Sexual partnerships are assumed to be ephemeral, and the mating dynamics occur as follows. The baseline per‐capita mating rate is *p*, which is independent of the population size, N=S+I. This means that larger populations do not have a higher per‐capita mating rate than smaller populations. Before deciding whether to mate, each host inspects its prospective partner for signs of infection (e.g., through visual or olfactory clues). If the prospective partner is currently uninfected, the probability that the focal host accepts the mate is $m_{S}\left(g\right)$, with $\frac{dm_{S}}{dg}\leq 0$. The function $m_{S}\left(g\right)$ allows for the fact that hosts may imperfectly assess the condition of other individuals and those who are choosier (higher *g*, lower $m_{S}\left(g\right)$) may be generally more cautious in their approach to mating, potentially declining healthy prospective partners. If the prospective partner is currently infected, the probability of accepting them as a mate is $m_{I}\left(g,v(\beta )\right)$ with $\frac{\partial m_{I}}{\partial g},\frac{\partial m_{I}}{\partial v}\leq 0$. By causing more damage to their hosts, more virulent STIs may be easier to detect by prospective mates (e.g., due to a general deterioration in health or more visible signs of infection). Preferential mating with uninfected hosts is somewhat comparable to the notion of disease causing lower contact rates (e.g., due to decreased movement) in classical evolution of virulence theory (Ewald 1983), although there are also a number of other notable differences in the present framework (sexual rather than direct transmission, and a reduction in contact rates affects reproduction).

Note that *g* is a dummy variable, which indicates the “strength of mate choice”, whereas $m_{S}\left(g\right)$ and $m_{I}\left(g,v(\beta )\right)$ are the actual probabilities of accepting an uninfected or infected prospective partner as a mate, respectively. I use a dummy variable for the strength of mate choice so that the host responses to susceptible and infected individuals are correlated. Throughout, it is assumed that the probability of accepting an uninfected individual as a mating partner is at least as large as the probability of accepting an infected individual: $m_{S}\left(g\right)\geq m_{I}\left(g,v(\beta )\right)$. Without this assumption, there would never be any advantages to mate choice, as choosier individuals would mate with infected members of the population at a higher rate than less choosy individuals. I therefore set $m_{I}\left(g,v(\beta )\right)=m_{S}\left(g\right){\tilde{m}}_{I}\left(g,v(\beta )\right)$ with ${\tilde{m}}_{I}\left(g,v(\beta )\right)\leq 1$ the mate choice response specific to prospective partners who are infected. In the analysis that follows, I set $m_{S}\left(g\right)=1-\zeta g$ for ζg<1 and 0 otherwise, where ζ is the cost of mate choice, and either ${\tilde{m}}_{I}\left(g,v(\beta )\right)=1-gv\left(\beta \right)$ (linear response) or ${\tilde{m}}_{I}\left(g,v(\beta )\right)=1-gv{\left(\beta \right)}^{2}$ (nonlinear response) with both functions restricted to ${\tilde{m}}_{I}\left(g,v(\beta )\right)\geq 0$. I only examine a linear function for $m_{S}\left(g\right)$ because *g* is a dummy variable and therefore one only needs to consider linear and nonlinear forms of one of the two correlated mate choice functions. Biologically, the linear and nonlinear functions for ${\tilde{m}}_{I}\left(g,v(\beta )\right)$ mean that the effects of mate choice either increase proportionately or accelerate with virulence. In other words, the linear function implies that the probability of accepting an infected mate is proportional to the damage caused by the STI, and the nonlinear function implies that hosts are disproportionately choosier when STIs are more virulent or that the STI is increasingly easier to detect. Although empirical evidence for how mate choice varies with virulence is currently lacking, it is plausible that either linear or nonlinear relationships could exist and therefore varying the shape of these functions is important for a better understanding of potential host‐STI coevolutionary dynamics.

The mating rates for each combination of the *S* and *I* classes are given by

(3) $$\;\left[SS\right]=\frac{pm_{S}{\left(g\right)}^{2}S^{2}}{N},$$

(4) $$\;\left[SI\right]=\frac{2pm_{S}\left(g\right)m_{I}\left(g,v\left(\beta \right)\right)SI}{N},$$

(5) $$\;\left[II\right]=\frac{pm_{I}{\left(g,v\left(\beta \right)\right)}^{2}I^{2}}{N}.$$

The factor of 2 in the equation for [SI] appears when there is mating between individuals in different classes and is required to balance the total mating rate, *M*:

(6) $$M=\left[SS\right]+\left[SI\right]+\left[II\right],$$

(7) $$\;=\frac{p{\left(m_{S}\left(g\right)S+m_{I}\left(g,v\left(\beta \right)\right)I\right)}^{2}}{N}.$$

Note that for the specific case when $m_{S}\left(g\right)=m_{I}\left(g,v(\beta )\right)=1$ (i.e., there is no mate choice), the total mating rate reduces to M=pN. For the general case, hosts that have mated produce offspring at a total rate of

(8) $$\begin{array}{ccc} b\left(f\left(\beta \right),g,v\left(\beta \right)\right) & = & pr\left(1-hN\right)\;(\left[SS\right]+f\left(\beta \right)\left[SI\right]\\ & & \;+f{\left(\beta \right)}^{2}\left[II\right]), \end{array}$$

(9) $$\;=\frac{pr\left(1-hN\right){\left(m_{S}\left(g\right)S+f\left(\beta \right)m_{I}\left(g,v\left(\beta \right)\right)I\right)}^{2}}{N},$$

where *r* is the maximum reproduction rate per pair and the birth rate is subject to density‐dependent competition given by the parameter *h*.

The disease‐free equilibrium $\left(S,I\right)=\left(S^{*},0\right)$ of this system occurs at

(10) $$\;S^{*}=\frac{1}{h}\left(1-\frac{d}{{\left(m_{S}\left(g\right)\right)}^{2}pr}\right)$$

and is viable provided ${\left(m_{S}\left(g\right)\right)}^{2}pr>d$ (i.e., the birth rate is higher than the death rate). A newly introduced STI will spread in a susceptible population when the basic reproductive ratio, $R_{0}\left(g,\beta \right)$, is greater than 1, where

(11) $$R_{0}\left(g,\beta \right)\;=\frac{2pm_{S}\left(g\right)m_{I}\left(g,v\left(\beta \right)\right)\beta }{d+\alpha \left(\beta \right)+\gamma }.$$

The above model describes the dynamics when there is only one host type and one STI type in the population. To account for situations where hosts vary in their strength of mate choice and STIs in the transmissibility/virulence, I adapt the above monomorphic model for populations that are polymorphic in these traits. The dynamics for $n_{h}$ hosts each with strength of mate choice $g_{i}$, where $i\in \{1,\ldots ,n_{h}\}$ and $n_{p}$ STIs each with transmissibility $\beta_{j}$ and virulence $v_{j}$ where $j\in \{1,\ldots ,n_{p}\},\;$are fully described by the following system of ordinary differential equations:

(12) $$\;\frac{dS_{i}}{dt}=b_{i}-\sum_{j}\beta_{j}\left[S_{i}I_{\circ j}\right]-dS_{i}+\sum_{j}\gamma I_{\mathrm{ij}},$$

(13) $$\;\frac{dI_{\mathrm{ij}}}{dt}=\beta_{j}\left[S_{i}I_{\circ j}\right]-\left(d+\alpha \left(\beta_{j}\right)+\gamma \right)I_{\mathrm{ij}},$$

where $\left[S_{i}I_{\circ j}\right]=\frac{2pm_{I}\left(g_{i},v_{j}\right)S_{i}}{N}\sum_{k}m_{S}\left(g_{k}\right)I_{kj}$, which is the total mating rate between susceptible hosts with trait $g_{i}$ and all hosts infected by STIs with traits $\beta_{j}$ and $v_{j}$, and the birth rate for each host type is

(14) $$\begin{array}{ccc} & & \;b_{i}=\frac{pr\left(1-hN\right)}{N}\left({\left[S_{i}S_{\circ }\right]}_{b}+{\left[S_{i}I_{\circ \circ }\right]}_{b}+{\left[S_{\circ }I_{i\circ }\right]}_{b}+{\left[I_{i\circ }I_{\circ \circ }\right]}_{b}\right)\;, \end{array}$$

where for notational convenience circles in subscripts correspond to sums over all host or parasite types (i.e., $S_{\circ }$ is the sum over all uninfected hosts, $I_{i\circ }$ is the sum over all hosts of type *i* that are infected, $I_{\circ j}$ is the sum over all hosts that are infected with parasite type *j*, and $I_{\circ \circ }$ is the sum over all infected hosts), so that

(15) $$\;{\left[S_{i}S_{\circ }\right]}_{b}=m_{S}\left(g_{i}\right)S_{i}\sum_{k}m_{S}\left(g_{k}\right)S_{k},$$

(16) $$\begin{array}{ccc} & & {\left[S_{i}I_{\circ \circ }\right]}_{b}=S_{i}\sum_{j}\left(f\left(\beta_{j}\right)m_{I}\left(g_{i},v_{j}\right)\sum_{k}m_{S}\left(g_{k}\right)I_{kj}\right), \end{array}$$

(17) $$\begin{array}{ccc} & & {\left[S_{\circ }I_{i\circ }\right]}_{b}=m_{S}\left(g_{i}\right)\sum_{j}\left(f\left(\beta_{j}\right)I_{\mathrm{ij}}\sum_{k}m_{I}\left(g_{k},v_{j}\right)S_{k}\right), \end{array}$$

(18) $$\begin{array}{ccc} & & {\left[I_{i\circ }I_{\circ \circ }\right]}_{b}=\sum_{j}\left(f\left(\beta_{j}\right)I_{\mathrm{ij}}\sum_{l}\left(f\left(\beta_{l}\right)m_{I}\left(g_{i},v_{l}\right)\sum_{k}m_{I}\left(g_{k},v_{j}\right)I_{kl}\right)\right). \end{array}$$

This model is related to the pair formation framework proposed in Ashby and Boots (2015), but there are two key differences. First, the model in Ashby and Boots (2015) assumes there is serial monogamy with separate pools of paired and unpaired individuals. Here, I focus on a single pool of individuals with ephemeral sexual partnerships, which is analogous to having infinite pair dissolution rates and instantaneous reproduction. However, it is not possible to move directly between the models by letting the pair dissolution rates tend to infinity, as this would mean individuals are never in the paired state and reproduction only occurs while individuals are paired. The second major difference is in the nature of the infection: the model in Ashby and Boots (2015) assumes STIs cause sterility rather than increase mortality, and that individuals are unable to recover once infected. Relaxing these assumptions, by allowing STIs to cause mortality and hosts to clear infection (as is the case for many STIs; e.g., Miyairi et al. 2010; Gizaw et al. 2017), will reveal how a broader range of STI life‐history traits affect coevolution with host mate choice. An additional difference in the current model is that the mate choice functions have been generalized to $m_{S}\left(g\right)$ and $m_{I}\left(g,v(\beta )\right)$ for easier interpretation and so that different functions governing mate choice can be readily explored.

I use evolutionary invasion analysis to determine the long‐term trait dynamics of the host and STI (Geritz et al. 1998). This assumes that mutations have small phenotypic effects, and for analytic rather than simulated solutions, mutations are sufficiently rare so that the system has reached a stable state before a new mutant emerges. For one‐sided adaptation (either host or STI evolution), I numerically solve the one‐dimensional fitness gradients to find the singular strategies, as the system is intractable to nonnumerical methods of stability analysis. For host‐STI coevolution, I solve the dynamics using simulations to capture nonequilibrium dynamics (i.e., fluctuating selection). In the coevolutionary simulations, host and STI traits are discretized into a finite number of different types and mutations between adjacent types occur at regular intervals (source code in the Supporting Information).

## Results

### PARASITE EVOLUTION

The invasion fitness of a rare mutant strain of the STI (subscript *m*) in a population at equilibrium $N^{*}=S^{*}+\;I^{*}$ is

(19) $$\begin{array}{ccc} w_{P}\left(g,\beta_{m}\right) & = & \frac{1}{I_{m}}\frac{dI_{m}}{dt}\;=\frac{2pm_{S}\left(g\right)m_{I}\left(g,v\left(\beta_{m}\right)\right)\beta_{m}S^{*}}{N^{*}}\\ & & -\left(d+\alpha \left(\beta_{m}\right)+\gamma \right)\;. \end{array}$$

The mutant STI can only invade when $w_{P}\left(g,\beta_{m}\right)>0$, which requires

(20) $$R_{\mathrm{EFF}}\left(g,\beta_{m}\right)=R_{0}\left(g,\beta_{m}\right)\left(\frac{S^{*}}{N^{*}}\right)>1,$$

where $R_{\mathrm{EFF}}\left(g,\beta_{m}\right)$ is the effective reproductive ratio of the STI. Because STI fitness can be written in this form, we know that parasite evolution maximizes its basic reproductive ratio *R*_0_ (Lion and Metz 2018). The STI will evolve in the direction of $\frac{\partial R_{0}}{\partial \beta }$ until β is maximized at 1, one or both populations are driven extinct, or a singular strategy $\beta^{*}$ is reached at $\frac{\partial R_{0}}{\partial \beta }{|}_{\beta =\beta^{*}}=0$, which requires

(21) $$\begin{array}{c} \;{\left.\frac{\partial m_{I}}{\partial \beta }\right|}_{\beta =\beta^{*}}=m_{I}\left(g,v\left(\beta^{*}\right)\right)\;\left(\frac{1}{d+\alpha \left(\beta^{*}\right)+\gamma }{\left.\frac{d\alpha }{d\beta }\right|}_{\beta =\beta^{*}}-\frac{1}{\beta^{*}}\right). \end{array}$$

**Figure 1:**
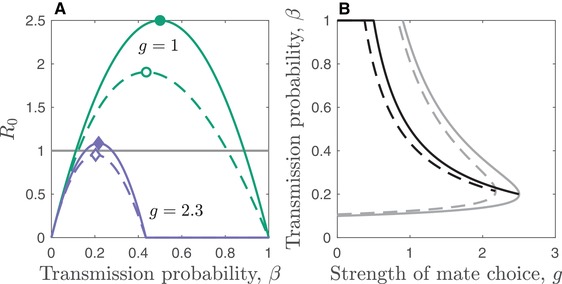
STI evolution in response to host mate choice for sterility (solid) and mortality (dashed) virulence. (A) The STI basic reproductive ratio, *R*_0_, for weaker (*g* = 1; green curves, circular markers) and stronger (*g* = 2.3; purple curves, diamond markers) mate choice. The horizontal line indicates the extinction threshold for the STI and the markers indicate the maximum value of *R*_0_. (B) Curves show the continuously stable strategies (CSSs, i.e., $\beta^{*}$) for a given strength of mate choice, g. The regions to the right and below the corresponding grey curves show where the STI is unviable. Mate choice and virulence functions as described in the text. Parameters: $m_{S}\left(g\right)=1$,γ=0.5, η=1, κ=1, d=1, $h=10^{-6},$ p=7.5, and r=20.

Because $m_{S}\left(g\right)$ does not feature in this equation, we do not need to consider the effects of costs of mate choice on STI evolution. In general, $m_{I}\left(g,v(\beta )\right)$ and f(β) will be decreasing (or constant) functions of β, and α(β) will be an increasing (or constant) function. In the absence of mate choice $\left(m_{S}\left(g\right)=m_{I}\left(g,v(\beta )\right)=1\right)$, a *continuously stable strategy* (CSS)—analogous to an *evolutionary stable strategy* or ESS—can only exist when α(β) is concave up (i.e., mortality virulence accelerates with the transmission probability). In the presence of mate choice, however, a CSS can exist under a broader set of conditions, such as concave down mortality‐transmission trade‐offs and with sterility‐transmission trade‐offs. This is clear from the equation for $R_{0}\left(g,\beta \right)$ (eq. 11), which features the product of $m_{I}\left(g,v(\beta )\right)$ and β (i.e., the product of decreasing and increasing functions of β; Fig. 1A).

To illustrate the above, suppose first that sterility virulence is constant $\left(\frac{df}{d\beta }=0\right)$. When there is no mate choice and mortality virulence is a linear function of β with α(β)=κβ, we have $\frac{\partial R_{0}}{\partial \beta }>0$, so the STI will evolve to maximize transmission at β=1 and virulence at α(1)=κ. If, however, mate choosiness is a linear function of mortality virulence (i.e., increasing damage has a linear effect on the probability of being accepted as a mate) such that v(β)=α(β)/κ and ${\tilde{m}}_{I}\left(g,v(\beta )\right)=1-\frac{g\alpha (\beta )}{\kappa }$ for gα(β)<κ and 0 otherwise, then a singular strategy exists at

(22) $$\;\beta^{*}=\frac{\sqrt{g\left(d+\gamma \right)\left(g\left(d+\gamma \right)+1\right)}-g\left(d+\gamma \right)}{g\kappa },$$

(Fig. 1B), which is always evolutionarily stable since

(23) $$\;{\left.\frac{\partial^{2}R_{0}}{\partial \beta^{2}}\right|}_{\beta =\beta^{*}}=-\frac{4gp\kappa m_{S}\left(g\right)}{\sqrt{g\left(d+\gamma \right)\left(g\left(d+\gamma \right)+1\right)}}<0.$$

Now suppose instead that mortality virulence is constant $\left(\frac{d\alpha }{d\beta }=0\right)$ and sterility virulence is a function of the transmission probability such that f(β)=1−ηβ, with v(β)=1−f(β). If host mate choosiness is a linear function of sterility virulence such that ${\tilde{m}}_{I}\left(g,v(\beta )\right)=1-g\left(1-f(\beta )\right)$, then the singular strategy occurs at $\beta^{*}=\frac{1}{2g\eta }$ (Fig. 1B), which again is always evolutionarily stable since

(24) $$\;{\left.\frac{\partial^{2}R_{0}}{\partial \beta^{2}}\right|}_{\beta =\beta^{*}}=-\frac{4\eta pm_{S}\left(g\right)\sqrt{3g}}{d+\gamma }<0.$$

All else being equal, the effects of mate choice on the ecology and evolution of the STI under sterility and mortality virulence are qualitatively similar (Fig. 1). However, because mortality virulence causes an additional reduction in *R*_0_ compared to sterility virulence due to the presence of α(β) in the denominator (eq. 11), a given level of mate choice will have a greater impact on an STI that causes mortality virulence. This can be seen in Figure 1, where both *R*_0_ and the evolved probability of transmission, $\beta^{*}$, of the STI are always lower under mortality virulence and the region of viability is smaller compared to STIs that cause sterility virulence.

In summary, host mate choice prevents the evolution of greater mortality or sterility virulence in an acute STI even in the absence of long‐term partnerships, but the effects on STIs that cause mortality virulence will tend to be greater, leading to lower disease prevalence and selection for slightly lower transmissibility for a given level of mate choice.

### HOST EVOLUTION

The initial dynamics of a rare mutant host (subscript *m*) in a resident population at equilibrium are given by

(25) $$\;\frac{dS_{m}}{dt}=b_{m}-\beta \left[S_{m}I^{*}\right]-dS_{m}+\gamma I_{m}$$

(26) $$\;\frac{dI_{m}}{dt}=\beta \left[S_{m}I^{*}\right]-\left(d+\alpha \left(\beta \right)+\gamma \right)I_{m}$$

with

(27) $$\begin{array}{ccc} \;b_{m} & = & \frac{p}{N^{*}}\left(r\left(1-hN^{*}\right)\left(m_{S}\left(g_{m}\right)S^{*}+f\left(\beta \right)m_{I}\left(g_{m},v\left(\beta \right)\right)I^{*}\right)\right.\\ & & \left.\left(m_{S}\left(g\right)S_{m}+f\left(\beta \right)m_{I}\left(g,v\left(\beta \right)\right)I_{m}\right)\right). \end{array}$$

Using the next‐generation method (see Supporting Information; Hurford et al. 2010), it can be shown that host fitness is sign‐equivalent to

(28) $$\begin{array}{ccc} & & w_{H}\left(g_{m}\right)\\ & & =\frac{prm_{S}^{g}\left(1-hN^{*}\right)\left(2pf_{\beta }m_{I}^{g,\beta }m_{I}^{g_{m},v_{\beta }}\beta I^{*}+\Gamma_{\beta }N^{*}\right)\left(m_{S}^{g_{m}}S^{*}+f_{\beta }m_{I}^{g_{m},v_{\beta }}I^{*}\right)}{N^{*}\left(2p\beta m_{S}^{g}m_{I}^{g_{m},v_{\beta }}I^{*}\left(\Gamma_{\beta }-\gamma \right)+d\Gamma_{\beta }N^{*}\right)}-1, \end{array}$$

where $\Gamma_{\beta }=d+\alpha \left(\beta \right)+\gamma$, $f_{\beta }=f\left(\beta \right)$, $m_{S}^{g}=m_{S}\left(g\right)$, and $m_{I}^{g_{m},v_{\beta }}=m_{I}\left(g,v(\beta )\right)$ for the sake of brevity. The host will evolve in the direction of$\;\frac{\partial w_{H}}{\partial g}$ until *g* is minimized at 0, one or both populations are driven extinct, or a singular strategy, $g^{*},$ is reached at $\frac{\partial w_{H}}{\partial g}{|}_{g=g^{*}}=0.$

**Figure 2:**
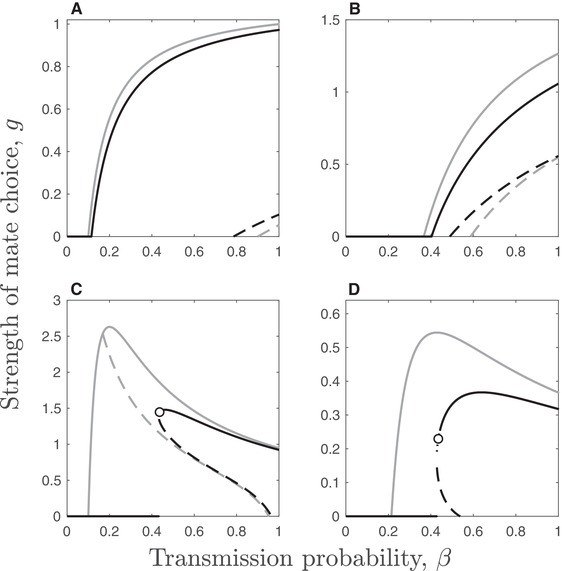
Evolution of the strength of host mate choice, *g*, in the presence of a nonevolving STI. Solid lines correspond to continuously stable strategies (CSSs), dashed lines to unstable strategies which act as evolutionary repellers, and circles to branching points, in the presence (black; ζ=0.1) and absence (gray; ζ=0) of host costs, with $m_{S}\left(g\right)=1-\zeta g$. The component of mate choice specific to infected individuals is as described in the text. Only one type of virulence (mortality or sterility) is assumed to occur in each panel with mate choice of infected individuals based on: (A) fixed sterility virulence, v(β)=0.9; (B) fixed mortality virulence, $\frac{v(\beta )}{\kappa }=0.5$; (C) variable sterility virulence, v(β)=1−f(β)=βη; and (D) variable mortality virulence, v(β)=α(β)=βκ. Remaining parameters as described in Figure 1, except κ=8.

Suppose initially that there are no costs of mate choice $\left(m_{S}\left(g\right)=1\right)$ and that mate choice is a linear function of virulence such that ${\tilde{m}}_{I}\left(g,v(\beta )\right)=1-gv\left(\beta \right)$. In this scenario, there may be one or two singular strategies. The singular strategy at $g_{1}^{*}=\left(2p\beta -d-\alpha (\beta )-\gamma \right)/2p\beta v\left(\beta \right)$ always exists, and corresponds to the point where the host drives the STI extinct. The second singular strategy $\left(g_{2}^{*}\right)$, if it exists, is an *evolutionary repeller* (i.e., a fitness minimum, so the direction of selection always points away from the singular strategy) with $0<\;g_{2}^{*}<g_{1}^{*}$, in which case the outcome depends on the initial conditions, with $g<g_{2}^{*}$ causing selection against mate choice, and $g>g_{2}^{*}$ leading to STI extinction due to mate choice (Fig. 2A, B).

If we first suppose that virulence is fixed (i.e., it does not vary with transmission, η=κ=0), mate choice is likely to evolve for intermediate transmission probabilities (Fig. 2A, B). When the probability of transmission is small, the STI is unable to spread even in the absence of mate choice $\left(R_{0}<1\right)$ and if the probability of transmission is close to 1, there may be selection against weak mate choice caused by the evolutionary repeller. This is because disease prevalence is high and so most attempted matings are with infected individuals, meaning that even weak mate choice dramatically reduces the mating rate for invading host mutants compared to the resident population. If, however, there is already a sufficient level of mate choice in the resident population (i.e., the initial conditions are above the repeller), disease prevalence is sufficiently low to allow runaway selection for mate choice, eventually driving the disease extinct. This pattern is similar regardless of whether virulence has fixed effects on mortality or sterility (Fig. 2A, B). When sterility or mortality virulence is linked to the transmission probability, the dynamics are more complex (Fig. 2C, D). Notably, the threshold for driving the STI extinct is lower at high transmission probabilities because virulence (and hence the effects of mate choice) are also stronger. An evolutionary repeller may exist, but it now occurs for intermediate values of β.

The system is intractable to classical analysis when mate choice also affects prospective partners that are susceptible (i.e., there is a “cost” of being choosy, with $m_{S}\left(g\right)<1$ for g>0), and so one must find the evolutionary dynamics using numerical analysis. Although many of the results are qualitatively similar to the no‐cost scenario, there are some notable exceptions. In particular, if the host evolves mate choice, then it no longer drives the STI extinct, and is instead likely to reach a CSS with the STI endemic in the population. When virulence is correlated with transmission, the host only evolves mate choice de novo at sufficiently high values of β (Fig. 2C, D). Additionally, there is a very small region of parameter space at intermediate values of β that can yield evolutionary branching, with stable coexistence between two host types: one that exhibits moderate mate choice and the other that does not discriminate against infected mates.

### COEVOLUTION

**Figure 3:**
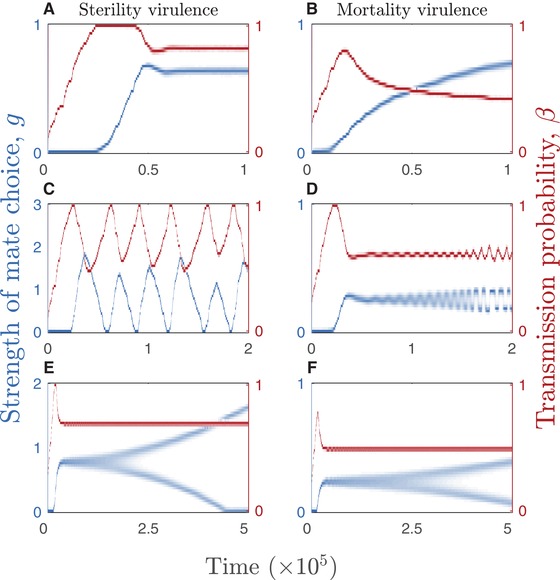
Coevolutionary dynamics between the strength of host mate choice, *g* (blue), and the STI transmission probability, β (red), under sterility (left column, v(β)=1−f(β)=ηβ) and mortality (right column, v(β)=α(β)/κ) virulence. (A–B) The host and STI evolve to co‐continuously stable strategies (co‐CSS). (C–D) The host and STI exhibit coevolutionary cycling (fluctuating selection). (E–F) The STI evolves to a continuously stable strategy (CSS) but the host branches into two strategies, one with high mate choice and the other with low/no mate choice. Fixed parameters: η=0.95, κ=8, d=1, $h=10^{-6},$ p=7.5, and r=20. Other parameters: (A) γ=0.5 and ζ=0.5. (B) γ=0.3 and ζ=0.03. (C) γ=0.5 and ζ=0.1. (D) γ=0.25 and ζ=0.15. (E) γ=3 and ζ=0.1. (F) γ=0.3 and ζ=0.075. Mating functions: (A) ${\tilde{m}}_{I}\left(g,v(\beta )\right)=1-gv\left(\beta \right)$. (B–F) ${\tilde{m}}_{I}\left(g,v(\beta )\right)=1-g{\left[v(\beta )\right]}^{2}$.

I now consider coevolution of the host and the STI, focusing on how the costs associated with mate choice (ζ), the recovery rate (γ), and the natural mortality rate of the host (*d*) interact with the mode of virulence (sterility or mortality) and the shape of the mate choice response to infected individuals. The model exhibits the same range of qualitative outcomes under both sterility and mortality virulence: (1) co‐CSS where STI virulence and host mate choice are at a stable equilibrium (Fig. 3A, B); (2) coevolutionary cycling, whereby host and STI phenotypes fluctuate through time (Fig. 3C, D); and (3) a stable level of virulence in the STI coupled with evolutionary branching in the host, where more and less choosy hosts are able to coexist (Fig. 3E, F). Note that the STI did not branch under any conditions.

**Figure 4:**
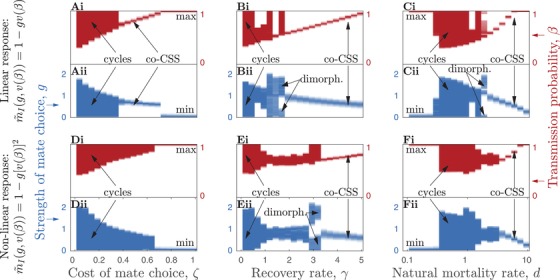
Qualitative and quantitative coevolutionary outcomes between the strength of host mate choice, *g* (blue), and the STI transmission probability, β (red), under sterility virulence (v(β)=1−f(β)=βη). Shading corresponds to the maximum frequency of each trait value over the final 2,000 evolutionary time steps (cycles are indicated by large solid regions). Panels A–C correspond to a linear mate choice function for ${\tilde{m}}_{I}\left(g,v(\beta )\right)$ and panels D–F to a nonlinear function of virulence. The outcomes are identified as maximization (max), minimization (min), co‐continuously stable strategies (co‐CSS), coevolutionary cycling (cycles), and dimorphism (dimorph.). (A and D) Effects of variation in the cost of mate choice, ζ. (B and E) Effects of variation in the recovery rate, γ. (C and F) Effects of variation in the natural mortality rate of the host, *d*. Fixed parameters as described in Figure 3, with: (A and D) γ=0.5; (B and C) ζ=0.1; and (C and F) γ=0.5 and ζ=0.1.

**Figure 5:**
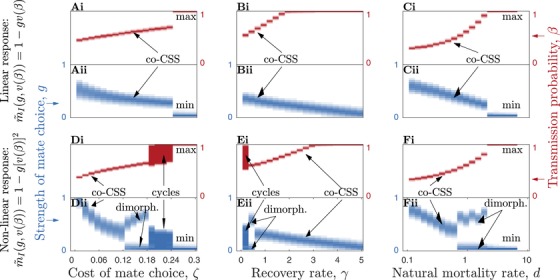
Qualitative and quantitative coevolutionary outcomes between the strength of host mate choice, *g* (blue), and the STI transmission probability, β (red), under mortality virulence (v(β)=1−α(β)/κ). Shading and labels as described in Figure 4. Fixed parameters as described in Figure 3, with: (A and D) γ=0.5; (B and E) ζ=0.15; (C and F) γ=0.5 and ζ=0.15.

The coevolutionary dynamics under sterility and mortality virulence are summarized in Figures 4 and 5 and Table 2. Overall, higher costs associated with mate choice (i.e., greater ζ leading to mistaken avoidance of uninfected individuals) tend to suppress choosiness and allow higher levels of virulence to evolve (Figs. 4A, D and 5A, D) and faster recovery rates have a stabilizing effect on the dynamics (Figs. 4B and 5B), as do both short (high *d*) and long (low *d*) host lifespans (Figs. 4C, F and 5C, F). Although both sterility and mortality virulence produce the same range of qualitative coevolutionary outcomes, there are some notable differences between the two scenarios. First, coevolutionary cycling is much more common under sterility virulence than mortality virulence, with the latter more likely to lead to stable equilibria. Even when mortality virulence does produce coevolutionary cycling, the amplitude of the cycles tends to be smaller compared to sterility virulence (Fig. 3C, D). Second, mate choice requires much lower costs (ζ) to evolve when the STI causes mortality virulence (Figs. 4A, D and 5A, D). Third, higher costs cause qualitatively different transitions in the coevolutionary dynamics, from cycling to stable strategies in the case of sterility virulence and from stable strategies to dimorphism and cycling in the case of mortality virulence (Figs. 4A and 5D). Fourth, although mate choice peaks for intermediate host lifespans in the case of sterility virulence (Fig. 4C, F), under mortality virulence mate choice generally decreases (or for a narrow window becomes dimorphic) as host lifespan shortens (as *d* increases; Fig. 5C, F). These general differences in outcomes are broadly consistent whether mate choice is linearly or nonlinearly related to virulence. Still, there are some notable differences in outcomes between the linear and nonlinear versions. For example, when greater virulence is associated with an acceleration in mate choice, there is usually a greater potential for coevolutionary cycling under sterility virulence (Fig. 4) and for coevolutionary cycling and evolutionary branching under mortality virulence (Fig. 5).

**Table 2:** Summary of the conditions that favor the different coevolutionary outcomes

	Sterility virulence	Mortality virulence
Stable strategies	Most likely for short/long lifespans, higher recovery rates, and intermediate/high costs.	Most likely for low costs, higher recovery rates, and short/long lifespans.
Coevolutionary cycling	Most likely for intermediate lifespans, low recovery rates, and low costs.	Only occurs for a narrow range of intermediate costs, with low recovery rates and intermediate lifespans.
Host branching (polymorphism)	Only occurs for a narrow range of intermediate costs, recovery rates, and lifespans.	Most likely for intermediate costs, low recovery rates, and intermediate lifespans.

## Discussion

Understanding the role of STIs in the evolution of host mating strategies, and in turn, the effects of mating behavior on disease evolution are inherently linked (Hamilton and Zuk 1982; Ashby and Boots 2015; Wardlaw and Agrawal 2019). Yet despite the large number of theoretical studies on coevolution between hosts and non‐STIs, to date theoretical models of STIs have almost exclusively focused on one‐sided adaptation rather than coevolution (Thrall et al. 1997, 2000; Knell 1999; Boots and Knell 2002; Kokko et al. 2002; Ashby and Gupta 2013; McLeod and Day 2014; Johns et al. 2019), which limits our ability to understand parasite‐mediated sexual selection.

Using a theoretical model of host‐STI coevolution, I have shown that host mate choice can readily evolve under a broad range of conditions, including when hosts have ephemeral sexual partnerships, STIs cause sterility or mortality virulence, hosts can recover from infection, and across large variations in host lifespan. In addition to showing when mate choice is most likely to evolve, I have also shown when qualitatively different coevolutionary outcomes typically occur (Table 2). Interestingly, coevolutionary cycling (fluctuating selection) in mate choice and STI virulence is much more common when the STI causes sterility virulence than mortality virulence, which may be because reductions in fecundity can cause sudden declines in population size and are generally known to induce oscillatory dynamics (Ashby and Gupta 2014). This suggests that STIs associated with higher mortality, such as dourine in equines (Gizaw et al. 2017), are more likely to lead to stable coevolutionary outcomes. But because STIs typically cause reductions in host fecundity (Lockhart et al. 1996), fluctuating selection may be a more probable outcome overall. Similarly, STIs often but not always cause chronic or long‐lasting infections, which will promote coevolutionary cycling because lower recovery rates tend to have a destabilizing effect. Higher clearance rates are associated with more stabilizing outcomes, so, for example, we might expect acute STIs such as *Chlamydia trachomatis* in mammalian species (Miyairi et al. 2010) to produce more stable coevolutionary dynamics than STIs with low or zero clearance rates. Furthermore, fluctuating selection dynamics appear to be limited to hosts with moderate rather than short or long lifespans. It is not entirely clear why fluctuating selection does not occur when host lifespans are taken to extremes, although it is likely to be related to changes in disease prevalence. Other factors may too vary with lifespan, but all else being equal, shorter host lifespans reduce the infectious period and hence disease prevalence and the risk of infection, whereas the converse is true for hosts with longer lifespans. Precisely what corresponds to an “intermediate lifespan” will clearly be system dependent, but overall, such predictions may help guide comparative analyses and other empirical studies toward systems that are more favorable to generating certain types of coevolutionary dynamics.

The model also revealed that evolutionary branching in host mate choice is possible, leading to the stable coexistence of more and less choosy individuals in the population. Hence if choosy individuals are above or below their equilibrium frequency, then they will decrease or increase in frequency, respectively. This only occurs when there are costs associated with being choosy, such that choosier individuals are not only less likely to mate with infected individuals but are also less likely to mate with susceptible individuals, for example, due to false‐positive detection of infection among healthy prospective partners. Hosts who are less choosy do not pay this cost but have a higher infection rate, which also increases disease prevalence in the population. This is analogous to trade‐offs with host life‐history traits that are often associated with resistance or tolerance to infection (Schmid‐Hempel 2003). Polymorphism in risky and prudent mating behavior has been identified previously by Boots and Knell (2002), although their model only allowed for variation in the overall mating rate rather than for condition‐dependent mate choice, and the mating rate for all infected individuals was the same regardless of whether hosts initially belonged to the risky or prudent mating type. Although the model presented herein can also generate polymorphism, this time in terms of host mate choosiness, it is only predicted to occur under a fairly narrow set of conditions, requiring moderate costs, recovery rates, and host lifespans.

A number of studies have looked at the evolution of host mating preferences in the presence of nonevolving STIs, showing, for example, that STIs can reduce mating skew provided disease prevalence is not too high (Kokko et al. 2002) and that STIs which cause mortality rather than sterility are more likely to drive the evolution of serial monogamy (McLeod and Day 2014). In general agreement with the present study, this body of theory predicts that STIs are likely to be a potent force of selection in host mating system evolution, and that factors such as the mode of virulence can lead to qualitatively different outcomes. Crucially, however, these studies do not account for reciprocal coevolution with the STI. Previous theoretical work on host‐STI coevolution appears to be limited to two models. A recent paper by Wardlaw and Agrawal (2019) explored the coevolution of hosts and STIs in the context of sexual conflict, showing that the mode of virulence (sterility or mortality) can lead to contrasting coevolutionary outcomes, analogous to the present study. Specifically, sterility virulence was shown to de‐escalate sexual conflict, whereas mortality virulence increased conflict, and furthermore led to an increase in STI virulence. The other study, Ashby and Boots (2015), is more closely related to the current model, as it too concerns the evolution of host mate choice for preferential mating with healthy hosts. The present study differs from this work both in terms of the mating dynamics (ephemeral sexual partnerships rather than serial monogamy) and the disease characteristics (acute infections causing sterility or mortality, rather than chronic sterilizing infections). Previously, it was unclear whether the qualitative coevolutionary outcomes such as fluctuating selection were restricted to the particular host and STI characteristics that were explored in Ashby and Boots (2015), but the current model reveals this not to be the case, and furthermore, predicts when these and other coevolutionary outcomes are most likely to occur. To build on these results, future work should continue to explore the evolution of mate choice under different mating systems, for example, for polygynous or polyandrous hosts, as different mating systems have previously been shown to select for contrasting levels of virulence (Ashby and Gupta 2013). One especially interesting direction for future research that would bridge the gap between the ephemeral partnership and serial monogamy scenarios would be to examine the evolution of mate choice when serially monogamous hosts engage in extra‐pair copulations (Forstmeier et al. 2014). This would help to elucidate whether mate choice should be stronger when choosing long‐ or short‐term mating partners.

Examining a broader set of STI characteristics helps to build up a more general picture of host‐STI coevolution, which is important given that many STIs do not cause chronic infections or sterility virulence (Lockhart et al. 1996; Miyairi et al. 2010; Gizaw et al. 2017). As discussed above, the mode of virulence has an important impact on the nature of the coevolutionary dynamics, with mortality virulence tending to have a stabilizing effect compared to sterility virulence. Overall, one might expect the benefits of mate choice to be greater if STIs cause sterility rather than mortality, as (1) individuals may be unable to reproduce following infection by a sterilizing STI but may still reproduce if infected by a nonsterilizing STI that increases mortality; and (2) disease prevalence is likely to be higher as mortality virulence reduces *R*_0_ by lowering the infectious period (eq. 11), which means all else being equal the risk of infection will be lower under mortality virulence. Recovery from infection will typically reduce the benefits of mate choice as both disease prevalence and the costs of contracting an infection are lower (since infection is acute rather than chronic). Recovery did not prevent the evolution of mate choice, but it did tend to have a stabilizing effect. As expected, high recovery rates reduce selection for mate choice, but surprisingly the difference in optimal levels of mate choice when infections are of relatively short or long durations is fairly small (Figs. 4B, E and 5B, E), which supports the notion that both acute and chronic STIs can select for mate choice. For simplicity, I assumed that recovery does not lead to immunity from future infection and that the condition of recovered individuals does not differ from those who have yet to experience infection. The former assumption is reasonable for many STIs, which are less likely than non‐STIs to result in lasting immunity (Lockhart et al. 1996), but the latter deserves further investigation.

Although it is possible for hosts to fully recover from infection, it is also reasonable to suspect that host condition may remain lower for some time following pathogen clearance, in which case these hosts should have lower mating success than individuals who have never been infected. In future, a simple extension of the current model would be to explore the effects of temporary or permanent reductions in host condition following infection, as this will separate the effects of mate choice into components representing transmission avoidance (i.e., avoiding infectious individuals) and partner fertility (i.e., choosing more fertile partners). Another potentially important extension to the current model would be to split the host population into males and females with different disease outcomes and transmission rates between the sexes. Here, the hosts were treated as a single hermaphroditic sex for the sake of simplicity, but such an extension would add greater realism and expand predictions for an even broader set of STIs with sex‐specific characteristics.

To date, many empirical studies have struggled to find evidence that hosts are able to discriminate between individuals with and without STIs (Abbot and Dill 2001; Webberley et al. 2002; Nahrung and Allen 2004). At first this seems surprising given that hosts should, in theory, be under strong selection to avoid choosing infected mates. There are a number of possible reasons as to why this may not always be the case. For example, hosts may simply be unable to detect signs of infection due to physiological constraints. This is not a particularly satisfying or general explanation, because various species have been found to prefer social or sexual partnerships based on visual or olfactory cues relating to infection (Clayton 1990; Willis and Poulin 2000; Moshkin et al. 2012). Instead, it is more likely that hosts may be unable to detect infection due to strong selection on STIs to be inconspicuous or asymptomatic, potentially through low virulence. For instance, sexually transmitted mites in ladybirds and the eucalypt beetle appear to have no negative impact on fertility or mortality under nonstress conditions, which may explain why mites do not appear to impact on mate choice in this system (Webberley and Hurst 2002; Nahrung and Clarke 2007; Ashby et al. 2019). It is also possible that STIs can evolve to be asymptomatic or difficult to detect despite being virulent, as is commonly the case with *Neisseria gonorrhoeae* infections (Gonnorrhea) in humans (Walker and Sweet 2011). More empirical evidence is needed to determine how STI virulence varies with detectability, whereas future theory needs to examine coevolution with virulent STIs, which may be asymptomatic and thus not easily detectable.

Another possibility is that hosts can sometimes discriminate between infected and uninfected individuals, but the costs of mate choice are too high relative to infection. In the current model, mate choice only evolves under certain conditions, and may not evolve even when the STI is relatively virulent, conspicuous, or prevalent, if mate choice is intrinsically costly. Clearly, any costs associated with mate choice (e.g., fewer mating opportunities) must be weighed against the potential benefits of avoiding infection. Alternatively, hosts may have more effective forms of defense against STIs, such as post‐copulatory grooming or urination to remove parasites (Hart et al. 1987; Nunn 2003). This area has received very little theoretical attention. Finally, it is possible that STIs cause changes in host characteristics such as attractiveness, mating frequency, or choosiness, which may potentially counter or increase selection for mate choice. In principle, such changes could simply be by‐products of infection or could be host or STI adaptations to increase fitness by achieving a higher mating rate, potentially leading to sexual conflict (Knell and Webberley 2004; Apari et al. 2014; Johns et al. 2019; Wardlaw and Agrawal 2019). The evolutionary consequences for STIs increasing mating rates have yet to be thoroughly explored and deserve much greater attention in future theoretical work. Similarly, the present model does not specify the cue for infection and whether this is via a sexually selected trait such as bright plumage that may affect the intrinsic attractiveness between individuals, but this would be a worthwhile distinction to explore theoretically. It is also worth noting that although the present study has focused on STIs, parasites that can be transmitted via other routes may also impact on mate choice (e.g., Clayton 1990), but theoretical models of mate choice have yet to consider coevolution with non‐STIs.

Given the general lack of predictions and data on host‐STI coevolution, there are clearly a number of important avenues for future theoretical and empirical research in this area. Still, the present study, combined with previous theoretical work (Ashby and Boots 2015; Wardlaw and Agrawal 2019), predicts that STIs and host mating systems are readily shaped by coevolutionary feedbacks. More specifically, this study has shown how mate choice and STI virulence are likely to coevolve under different host and STI life‐history traits, including variation in the mode of virulence, recovery rates, and host lifespan. Together, these results suggest that parasite‐mediated sexual selection is likely to select for mate choice under a broad set of conditions.

## CONFLICT OF INTEREST

The author declares there is no conflict of interest.

Associate Editor: K. Koelle

Handling Editor: D. W. Hall
