# Supplementary material for: Antagonistic coevolution between hosts and sexually transmitted infections
Source: Evolution. 2019 Dec 17;74(1):43–56. doi: 10.1111/evo.13883 (PMC6973023; doi:10.1111/evo.13883)
Supplement: Supplementary file 1 [file EVO-74-43-s001.pdf]

Supporting information:
Antagonistic coevolution between hosts and sexually transmitted infections

DERIVATION OF HOST FITNESS

As described in the main text, the initial dynamics of a rare mutant host (subscript m) in a resident population at equilibrium are given by:

$$\frac{dS_m}{dt} = b_m - \beta[S_m I^*] - dS_m + \gamma I_m \quad (S1)$$

$$\frac{dI_m}{dt} = \beta[S_m I^*] - (d + \alpha(\beta) + \gamma) I_m \quad (S2)$$

with

$$b_m = \frac{p}{N^*}\left(r(1 - hN^*)(m_S(g_m)S^* + f(\beta)m_I(g_m, v(\beta))I^*)(m_S(g)S_m + f(\beta)m_I(g, v(\beta))I_m)\right) \quad (S3)$$

corresponding to the birth rate and

$$\beta[S_m I^*] = \frac{2\beta p m_S(g) m_I(g_m, v(\beta)) S_m I^*}{N^*} \quad (S4)$$

to the infection rate.

One can use the next-generation method to calculate the invasion fitness for the rare mutant (Hurford et al. 2010), which first requires deriving the Jacobian of the mutant dynamics:

$$J = \begin{pmatrix} A - \frac{2\beta p m_S(g) m_I(g_m, v(\beta)) I^*}{N^*} - d & B + \gamma \\ \frac{2\beta p m_S(g) m_I(g_m, v(\beta)) I^*}{N^*} & -d - \alpha(\beta) - \gamma \end{pmatrix} \quad (S5)$$

where for notational convenience:

$$A = \frac{p m_S(g) r(1 - hN^*)(m_S(g_m)S^* + f(\beta)m_I(g_m, v(\beta))I^*)}{N^*} \quad (S6)$$

$$B = \frac{p m_I(g, v(\beta)) r(1 - hN^*)(m_S(g_m)S^* + f(\beta)m_I(g_m, v(\beta))I^*)}{N^*} \quad (S7)$$

Next, one must separate the Jacobian into components, F and V such that $J = F - V$ with:

$$F = \begin{pmatrix} A & B \\ 0 & 0 \end{pmatrix} \tag{S8}$$

$$V = \begin{pmatrix} \frac{2\beta p m_S(g) m_I(g_m, v(\beta)) I^*}{N^*} + d & -\gamma \\ -\frac{2\beta p m_S(g) m_I(g_m, v(\beta)) I^*}{N^*} & d + \alpha(\beta) + \gamma \end{pmatrix} \tag{S9}$$

The next-generation matrix is given by $N_G = FV^{-1}$, and the invasion fitness of the rare host mutant, $w_H(g_m, \beta)$, is sign equivalent to the largest eigenvalue of this matrix minus 1:

$$w_H(g_m) = \frac{prm_S^g(1 - hN^*)\left(2pf_\beta m_I^{g,\beta} m_I^{g_m,v_\beta} \beta I^* + \Gamma_\beta N^*\right)\left(m_S^{g_m} S^* + f_\beta m_I^{g_m,v_\beta} I^*\right)}{N^*\left(2p\beta m_S^g m_I^{g_m,v_\beta} I^*\left(\Gamma_\beta - \gamma\right) + d\Gamma_\beta N^*\right)} - 1 \tag{S10}$$

where $\Gamma_\beta = d + \alpha(\beta) + \gamma$, $f_\beta = f(\beta)$, $m_S^g = m_S(g)$ and $m_I^{g_m,v_\beta} = m_I(g, v(\beta))$, as shown in equation 31 in the main text.

REFERENCES

Hurford, A., D. Cownden, and T. Day. 2010. Next-generation tools for evolutionary invasion analyses. J. R. Soc. Interface 7:561–571.
